# Action observation activates neurons of the monkey ventrolateral prefrontal cortex

**DOI:** 10.1038/srep44378

**Published:** 2017-03-14

**Authors:** Luciano Simone, Marco Bimbi, Francesca Rodà, Leonardo Fogassi, Stefano Rozzi

**Affiliations:** 1 Department of Neuroscience, University of Parma, via Volturno 39, 43125 Parma, Italy; 2 Dipartimento di Scienze biomediche e chirurgico specialistiche, Università di Ferrara, via Fossato di Mortara 64/A 44121, Ferrara, Italy

## Abstract

Prefrontal cortex is crucial for exploiting contextual information for the planning and guidance of behavioral responses. Among contextual cues, those provided by others’ behavior are particularly important, in primates, for selecting appropriate reactions and suppressing the inappropriate ones. These latter functions deeply rely on the ability to understand others’ actions. However, it is largely unknown whether prefrontal neurons are activated by action observation. To address this issue, we recorded the activity of ventrolateral prefrontal (VLPF) neurons of macaque monkeys during the observation of videos depicting biological movements performed by a monkey or a human agent, and object motion. Our results show that a population of VLPF neurons respond to the observation of biological movements, in particular those representing goal directed actions. Many of these neurons also show a preference for the agent performing the action. The neural response is present also when part of the observed movement is obscured, suggesting that these VLPF neurons code a high order representation of the observed action rather than a simple visual description of it.

A traditional role attributed to prefrontal cortex is that of using contextual information for selecting and planning appropriate behavioral responses. Among contextual cues, particularly important for behavioral planning in nature is information about the social environment. The actions performed by other individuals and the identity of the agents of these actions are the most relevant sources of this type of information. Most of the data available in the literature on ventrolateral prefrontal cortex (VLPF) are about the coding of others’ identity, indicating that this cortical sector contains neurons responding to observation of faces, either static^1^,^2^; or associated to vocalization^3^,^4^. These neural responses probably rely on the presence of neuroanatomical connections between VLPF and areas belonging to the superior temporal sulcus^5^,^6^,^7^ whose neurons are known to respond to biological stimuli, including faces^8^,^9^,^10^. On the contrary, it is virtually unknown whether prefrontal neurons code observed actions. An indirect suggestion that these neurons may exist derives from the evidence of neuroanatomical connections of VLPF with inferior parietal, ventral premotor and inferior temporal cortex^7^,^11^,^12^,^13^,^14^,^15^. These regions form a network involved in action observation (see also ref. 16). In particular, in STS there are neurons activated during observation of body postures, locomotion and forelimb movements^17^,^18^,^19^,^20^,^21^, and in the parietal and premotor cortex there are neurons activated during observation of goal-directed hand actions^22^,^23^,^24^,^25^.

In the light of the above considerations, it becomes relevant to verify whether also VLPF contains neurons responding to action observation. To this purpose, we trained monkeys to observe different types of videos showing biological movements and object motion and contemporarily recorded the neuronal activity from VLPF. Biological movements included both goal-directed actions performed by monkeys or humans and non-goal-directed movements performed by humans. The main result of the study is that there are neurons responding to biological movements and that, among them, the highest percentage prefers goal-directed actions, in particular those performed by monkeys.

These results raise the important issue of the type of format in which VLPF neurons code the observed action. In order to address this issue, we devised a control task in which different parts of the videos were obscured. The results of this task revealed that the response of most of the tested neurons was not affected by this manipulation. We propose that these VLPF neurons code a high order representation of the observed action rather than a simple visual description of it.

## Results

**Figure 1:**
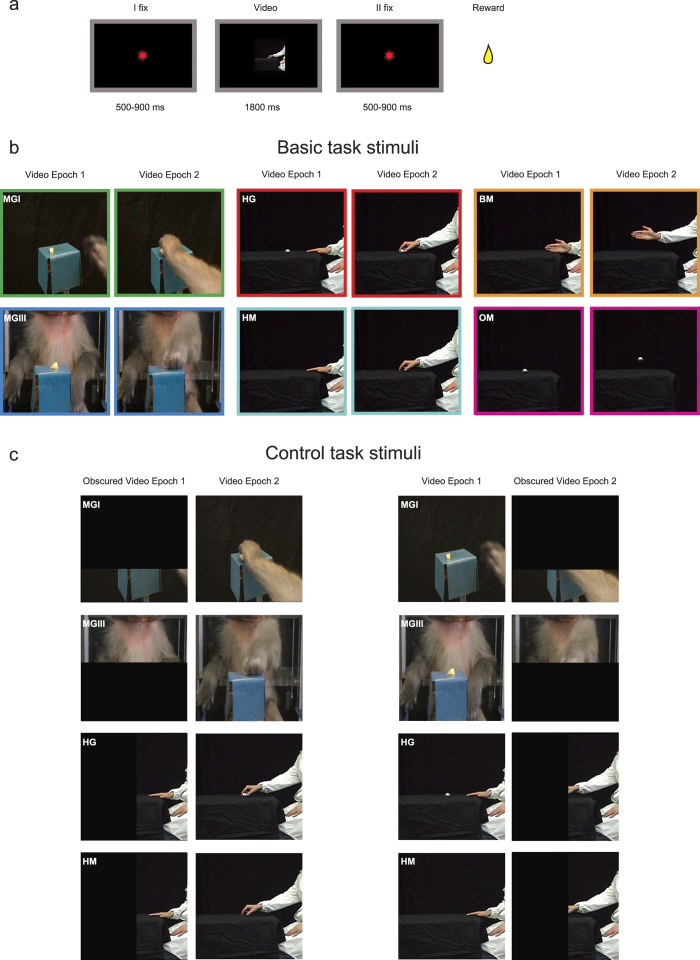
(**a**) Temporal sequence of events in the behavioral paradigms. (**b** and **c**) Representative frames taken from Video Epochs 1 and 2 of the video clips presented in the basic task and in the control task, respectively. MGI: Monkey grasping in first person perspective; MGIII: Monkey grasping in third person perspective; HG: Human grasping; HM: Human mimicking; BM: biological motion; OM: object motion. Each video covered an angle of 12 × 12° centered on the fixation point.

In order to evaluate the response of VLPF neurons to the observation of biological movements and object motion, we devised a task in which monkeys were required to keep their hand (contralateral to the recorded hemisphere) on a resting position and to observe different videos presented on a video monitor (see Materials and Methods). The sequence of events occurring during each trial is shown in Fig. 1a. The videos employed (Fig. 1b) depicted a monkey grasping a piece of food seen from a first (Monkey Grasping I, MGI) or third (Monkey Grasping III, MGIII) person perspective, a human actor, seen from a lateral view, grasping an object (Human Grasping, HG), mimicking this action (Human Mimicking, HM) or simply extending his forelimb in front of himself (Biological movement, BM), and the motion of an object (Object Motion, OM).

### Observation of fully visible actions

We recorded the activity of 584 VLPF task-related neurons, that is, those showing a significant modulation of their discharge during at least one of the epochs (Video epoch I and/or II) of at least one of the tested videos relative to baseline (3 × 6 ANOVA for repeated measures, p < 0.01, see Materials and Methods). Most neurons (n = 482) did actually respond significantly during the presentation of the video stimuli without showing any selectivity for the type of stimulus (only main effect), while 102 (17.5%) showed interaction effects (p < 0.01) revealing different types of visual selectivity. If these neurons are categorized according to the number of effective videos eliciting their discharge, the majority (n = 66) responded to only one video, 16 activated for two videos, 8 for three videos, 8 for four videos, and 4 neurons responded to five videos.

Based on the results of post-hoc tests, 77 out of 102 neurons (75.5%) were defined as Highly Selective because discharged stronger for only one video (HS neurons; interaction effect p < 0.01, and post-hoc revealing that one or both video epochs of a specific video is/are significantly different from its baseline and from that/those of all other videos). Most of them (n = 66) responded exclusively to one of the tested video stimuli (in addition to the definition of HS, in none of the other five videos any video epoch is significantly different from baseline), while 11, although responding to more than one video, exhibited a stronger discharge for one of them. The remaining 25, non-HS, neurons (24.5%) responded with a similar discharge to more than one of the video stimuli (interaction effect p < 0.01, and post-hoc revealing that: a) one or both video epochs of more than one video is/are significantly different from their baseline and from that/those of the remaining videos, and b) no significant difference is present in neural discharge among the effective videos).

**Figure 2:**
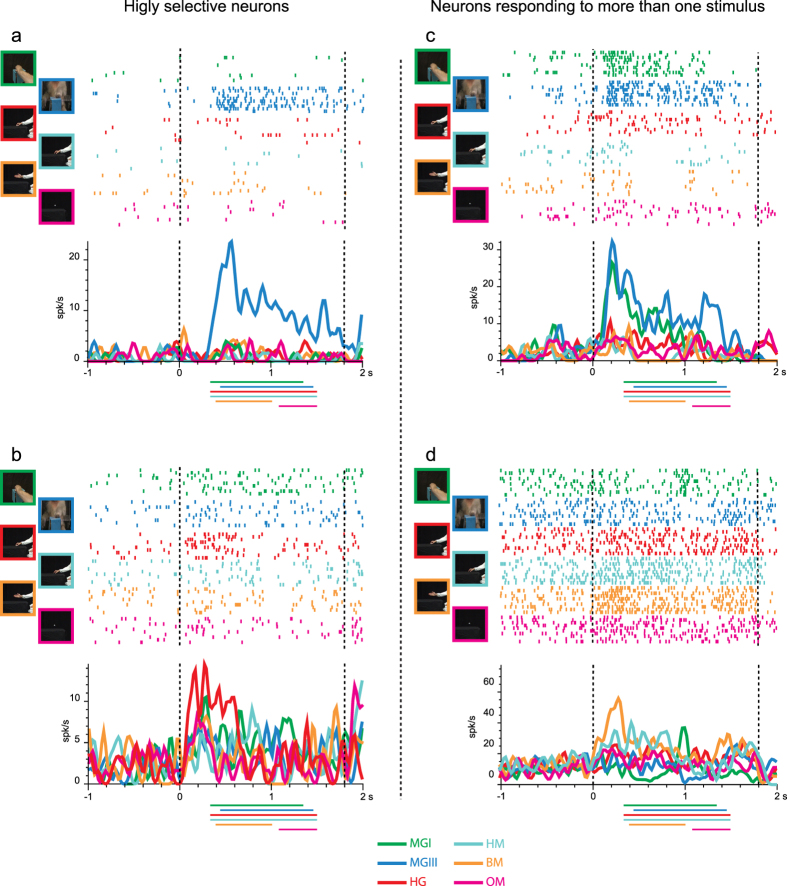
Examples of VLPF neurons responding during the observation of videos: highly selective neurons (**a**,**b**) and neurons responding to more than one video (**c**,**d**). (**a**) Neuron responding exclusively during the observation of a monkey grasping a piece of food from a third person perspective. (**b**) Neuron responding exclusively to a human actor grasping an object. (**c**) Neuron responding during the observation of a monkey grasping food from both the first and third person perspective. (**d**) Neuron responding during the observation of a human agent moving his arm in front of himself or mimicking a grasping action The vertical dashed lines indicate the beginning and the end of the video presentation. The activity is aligned on the beginning of the video presentation. Colored lines under histograms indicate the duration of movement in the corresponding video. Abscissae: time (s); Ordinates: firing rate (spikes/s).

Examples of HS neurons are shown in Fig. 2. Figure 2a shows the discharge of a neuron responding only during the observation of a monkey grasping a piece of food from a third person perspective (MGIII). The discharge begins at movement onset, peaks just before hand-object interaction and continues until the end of the video. Figure 2b shows the response of a neuron discharging stronger to the observation of a human actor grasping an object (HG). The neuron starts firing at video onset when movement has not started yet and discharge till hand-object interaction. Figure 2c and d shows examples of neurons responding to more than one stimulus. The neuron depicted in Fig. 2c responds during observation of a monkey grasping food from both the first and third person perspective. The peak of discharge occurs before movement onset and the discharge is sustained, although decreasing, till the end of the movement. The neuron shown in Fig. 2d responds during the observation of a human agent extending his arm in front of himself (BM) or mimicking a grasping action (HM). It does not respond to videos showing a human actor or a monkey grasping, or to the observation of object motion. The strongest discharge for BM is present before movement onset, while for HM the discharge starts before movement onset, unfolds during reaching movement and decreases in the lifting phase.

Thus, considering the effective videos, it seems that also neurons responding to more than one stimulus, nonetheless code specific aspects of the videos (e.g. type of agent, non-goal related movements or absence of objects).

In order to assess the degree of selectivity of the recorded neurons we calculated two indexes, one representing the difference between the maximal and the minimal response normalized to the cell’s maximal response (depth of tuning index, d_i_), the other the extent to which activity in all non-preferred stimuli deviates from maximal activity (selectivity index, s_i_) (see Methods;^26^). A d_i_ of 1 means that a neuron does not fire at all above baseline in the worst condition, a s_i_ of 1 means that a neuron responds only to one video stimulus. A d_i_ or an s_i_ of 0 indicates that the firing rate is the same for all videos. This latter situation, however, is absent in our dataset, since we included only neurons having different degrees of selectivity. Thus, both indexes are always higher than 0.

**Figure 3:**
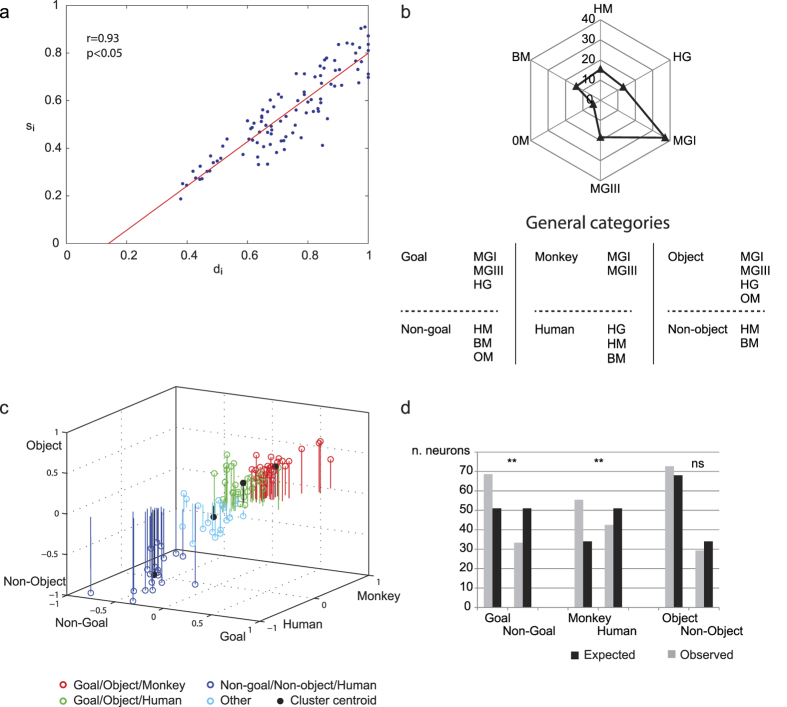
(**a**) Correlation between the Depth of tuning (d_i_) and Selectivity (s_i_) indexes. Each dot represents a neuron. (**b**) *Top.* Polar plot representing the number of neurons responding to each video stimulus. Each neuron is counted as 1 if it responds only to one stimulus, while when it responds to more than one stimulus, is counted as 1 divided by the number of effective stimuli. *Bottom.* Subdivision of the video stimuli in three pairs of general categories. (**c**) Plot of the normalized neural discharge according to the category indexes, represented in the three axes: Goal/Non-Goal; Monkey/Human; Object/Non-object. Values around zero on one axis correspond to low selectivity in that specific index. Colors indicate the four different clusters. (**d**) Histograms representing the expected and observed numbers of neurons belonging to the three pairs of general categories as defined in (**b**). Asterisks indicate a significant difference (p < 0.01) between expected and observed numbers.

In Fig. 3a, individual neurons are plotted according to their corresponding value in the two indexes. It is clear that most neurons show a high degree of preference in both indexes, being located in the upper right part of the plot. Note also that the correlation between the two indexes is very high (r = 0.93, p < 0.05), indicating that the highest the difference between the discharge to the best and the worst stimulus, the highest the neuron selectivity for a limited number of stimuli.

Concerning the preference for the type of video stimulus, HS neurons were classified on the basis of the stimulus producing the strongest response (Table 1). The most often coded stimuli were those representing a monkey grasping an object in first or third person perspective, followed by those representing a human subject grasping an object, mimicking this motor act or moving his arm. Finally, the responses specific to object motion were very rare (n = 2), suggesting that the presence of an agent is crucial for eliciting the discharge of this group of neurons.

The choice of the set of video stimuli was prompted by the idea to group them on the basis of more general categories, such as goal-relatedness, type of agent and presence of an object. Other more specific aspects, such as type of grip or hierarchical rank of the observed agent, were not included in our choice in order to limit the variables to be investigated and concentrate on more general aspects. Figure 3b indicates the number of neurons responding to each video stimulus, and the types of video stimuli belonging to each general category. In order to evaluate whether the whole population of neurons revealing some visual selectivity could fall into these different categories, for each excitatory neuron we plotted the index representing, in a 3D space, the relatedness to the three pairs of the above mentioned categories (Fig. 3c, see Methods). Neurons separate into four clusters (K-means analysis; mean silhouette value: 0.62, see Methods): (1) goal-directed actions performed by monkeys; (2) goal-directed actions performed by humans; (3) non-goal related actions; (4) neurons not showing a clear preference in at least one of the investigated categories, falling in a sector close to zero. These data indicate that the large majority of these VLPF neurons fall in one of the general categories.

Figure 3d shows the comparison between the expected and observed number of neurons belonging to the three pairs of categories, on the null hypothesis that each video stimulus is equally coded by prefrontal neurons. It is clear that this hypothesis is rejected because there is a significant imbalance in favor of goal-related (χ^2^ = 12.17072, p < 0.01) and monkey-related videos (χ^2^ = 15.00748, p < 0.01). On the contrary, absence vs presence of object does not seem to be differentially coded by these neurons (ns).

Concerning the phase of the video, we calculated the epoch preference of HS neurons. Among them, the majority (n = 39) responded during Video epoch 2, while 25 neurons responded during Video epoch 1. Thirteen neurons discharged equally well in both epochs. Neurons selective for Epoch 2 or responding in both epochs likely code either specific aspects of the observed movement (e.g. hand-object interaction) or the whole action. Neurons selective for Epoch 1 could also code the context or predict action beginning and its outcome. This latter hypothesis is not unlikely, since the context gives enough cues to the monkey to make such predictions. This is in line with the known properties of prefrontal neurons to exploit contextual information for planning and guiding behavior.

Interestingly, comparing the phases of the videos displaying grasping actions performed by the monkey in different perspectives, it emerges that while the responses to the monkey presented in third person show a slight preference for Video epoch 1 (epoch 1: n = 5; epoch 2: n = 2; epochs 1 and 2: n = 8), the majority of the responses to the monkey presented in first person perspective exhibited their preference during the second epoch (epoch 1: n = 9; epoch 2: n = 21; epochs 1 and 2: n = 1).

Seventy-seven neurons were also tested for their motor properties in a reaching-grasping task (see ref. 27). Of them 9 (12%) responded during the execution of reaching-grasping actions. Interestingly, all these latter neurons responded to the observation of at least one stimulus depicting goal directed actions. This response strictly resembles that described for the mirror neurons recorded in both ventral premotor and inferior parietal cortex.

### Observation of masked actions

In order to test which properties of the stimuli were crucial to elicit the neural responses described above, we employed a modified version of the actions observation task. In this task either the first or the second phase of the videos showing goal directed or mimicked actions could be partially obscured (see Materials and Methods). The neuron response recorded during the observation of the masked action was compared with that obtained during the observation of the non-masked stimuli. Note that masked and non-masked stimuli were presented in the same session.

Thirty-six neurons out of those responding to at least one between HG, HM, MGI and MGIII videos were tested in the control task. Based on their responses in the basic observation task, 21 neurons were tested with the videos showing monkey actions (either MGI, MGIII or both), 14 with those depicting human grasping and mimicking (either HG, HM or both), and one with both types of videos.

**Figure 4:**
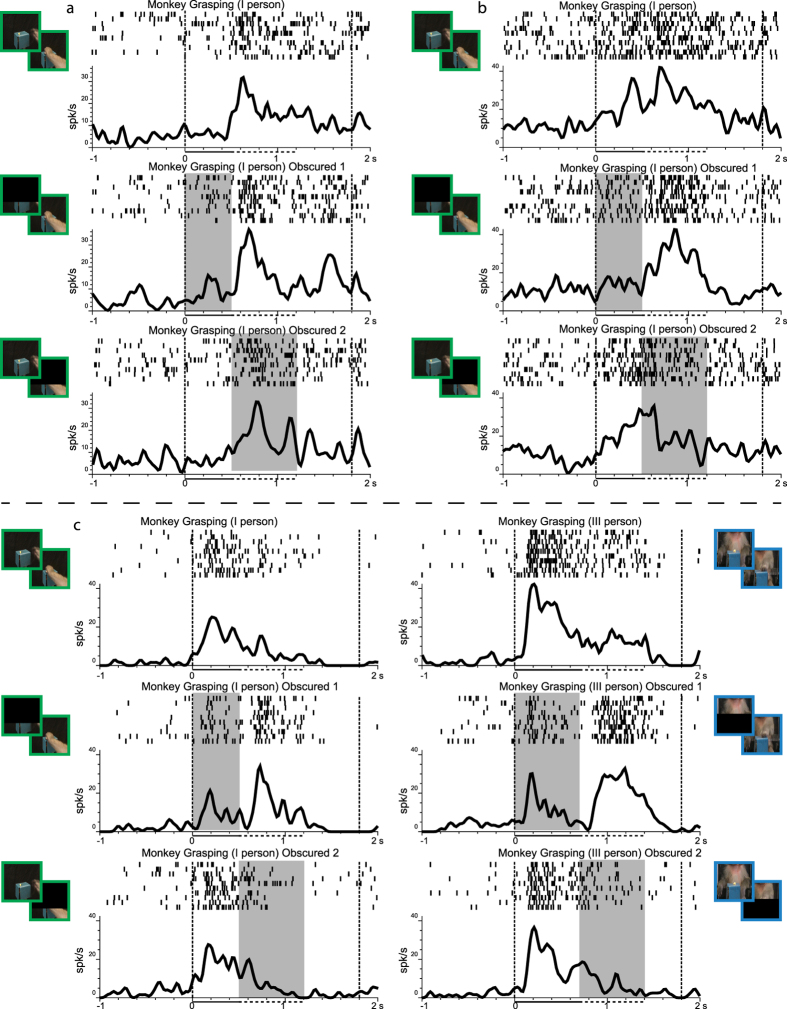
(**a**) Example of a neuron discharging during the observation of a monkey grasping an object from first person perspective (Video Epoch 2), that, with respect to when the video is fully visible (top), does not change its discharge when either the first (middle) or second (bottom) video epoch is obscured. (**b**) Example of a neuron that in the basic condition (top) discharges during both epochs of the video depicting a monkey grasping from a first person perspective. The response diminishes in each epoch when obscured. (**c**) Example of neuron discharging during the observation of a monkey grasping from a first (left) and third (right) person perspective. In the basic condition (top), the neuron shows its strongest response in the first epoch of both videos. When the first part of the videos is obscured, the discharge shifts from the first to the second epoch while the masking of the second part of the video does not affect the response shown in the basic condition. The shaded areas on rasters and histograms indicate the timing of obscuration. The horizontal lines under the x axis indicate the duration of the first (continuous line) and second (dashed line) Video Epoch, respectively. Other conventions as in Fig. 2.

The results of the 3 × 2 ANOVA (see Methods) show that 24 neurons did not change their activity when the video was obscured either in the first or second phase (Interaction effect not significant). Figure 4a shows an example of this neural behavior. The neuron, selective for MGI, responds during the second phase of the video in the basic condition and in the two obscured conditions, thus indicating that these neurons do not code purely visual aspects of the videos. Twelve neurons changed their discharge during the occlusion of part of the video. In particular, 3 neurons increased and 4 decreased their firing rate during obscuration of the epoch in which they showed the strongest discharge in the basic condition. Figure 4b shows an example of a neuron that in the basic condition discharges during both phases of the video (MGI). The response clearly decreases during video Epoch1, when the first part of the video is occluded, and during video Epoch 2, when the second part of the video is occluded, suggesting that visual features are crucial for activating this neuron. Interestingly enough, 5 neurons that in the basic condition discharged in the first epoch of the video, during obscuration of this epoch decreased their firing rate, but begun to fire in the second epoch. An example of one of these neurons is shown in Fig. 4c. This neuron, responding stronger in Epoch1 to the observation of a monkey grasping in first (left column) and third (right column) perspective, decreases its discharge when this epoch is obscured and increases it in Epoch2.

### Anatomical localization of the recorded neurons

**Figure 5:**
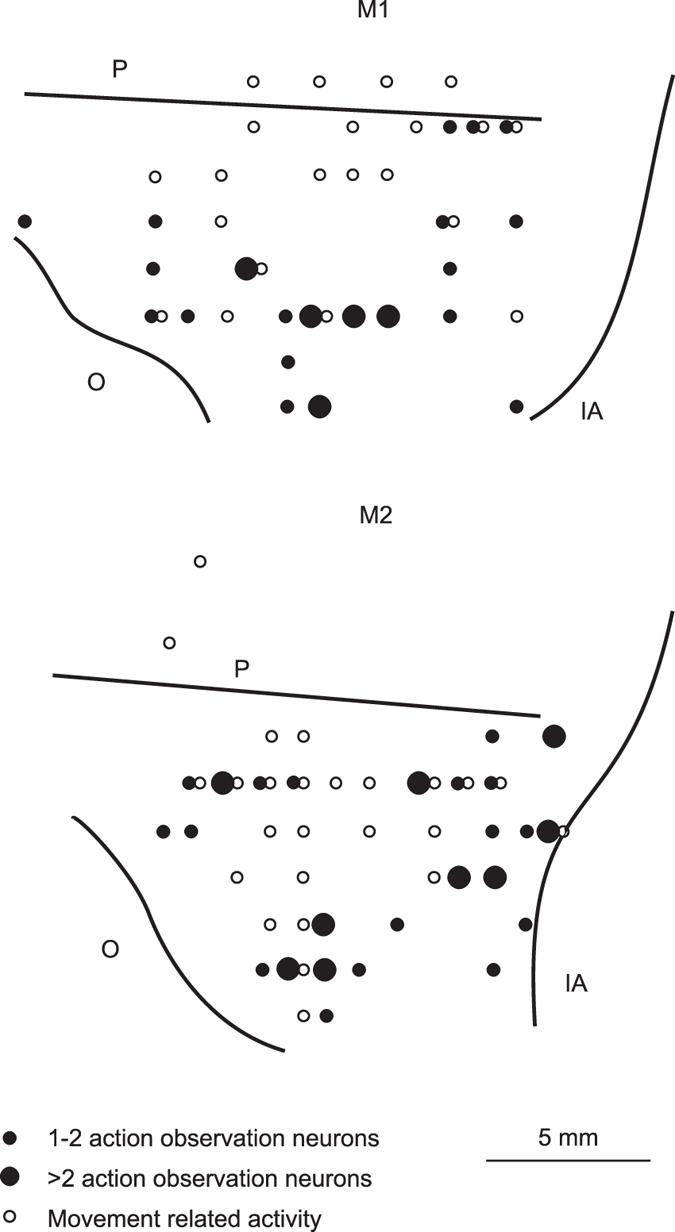
Functional reconstruction of the recorded region. Distribution of penetrations containing video selective neurons (filled circles) in the recorded region of the two monkeys (M1 and M2). The empty circles represent the penetrations in which we recorded movement-related neurons in a previous study in the same monkeys^27^. The position of the sulci is based on the penetrations depth. IA: inferior limb of the arcuate sulcus; O: orbital reflection; P: principal sulcus.

Figure 5 depicts the functional reconstruction of the recorded region of each monkey. The recorded sector covers a large cortical region, including most of VLPF, excluding its rostralmost part, and slightly extends into the dorsolateral prefrontal cortex (DLPF). Neurons responding to video presentations were found in penetrations (filled circles, 21 in M1 and 25 in M2) widely distributed in the recorded region, extending over the location of architectonic areas 12, 45 and 46. We observed no clear segregation in the distribution of neurons responding to different videos or categories of videos, or between HS and non-HS. The empty circles represent the penetrations in which we recorded movement-related neurons in a previous study in the same monkeys^27^. Note that there is a partial overlap between the location of the neurons responding during the observation of videos representing actions and during action execution.

## Discussion

The results of the present study show that in VLPF there are neurons responding to observation of biological movements performed with the forelimb. The majority of them showed a stimulus-specific activity, responding best or exclusively to one of the presented stimuli (HS). The highest percentage of HS neurons presented their strongest discharge during observation of goal-directed actions: the most effective were those performed by a monkey, while the human goal-directed action was coded by a lower number of neurons. The discharge of the majority of neurons was not affected when part of the observed action was obscured. Among the neurons whose discharge was affected by the obscuration of the first epoch, some shifted their discharge to the second, fully visible one. Finally, 12% of VLPF neurons tested for motor properties, beside discharging during hand action observation, discharged also when the monkey performed a grasping action.

### VLPF neurons do not respond to simple visual features of the video stimuli but likely encode specific categories

The neuronal responses to videos found in this study could be interpreted, in principle, as responses to specific visual features. We can exclude this explanation for several reasons. First of all, in spite of the fact that different videos share some visual features, most neurons belong to the HS category, thus by definition cannot respond to common visual features. Then, considering the visual features that could play a role in determining neuronal specificity, the most relevant are size and spatial location of the moving effector, that vary among different videos. However, if these were the coded variables, one would have expected to find neurons responding equally to stimuli of the same size, or coming from the same side of the video (i.e. Human grasping, Human mimicking and Biological movement). Only one neuron showed this behavior. Moreover, if neurons mainly coded visual features, the obscuration of part of the video should always dramatically decrease the neuronal response. This was the case only in about ten percent of the neurons tested with this control task. Altogether, these arguments indicate that a purely sensory explanation of the neural responses cannot account for the type of coding of the recorded VLPF neurons.

In agreement with the above observations, it is commonly accepted that the sensory responses of prefrontal neurons do not simply reflect specific sensory features^28^,^29^,^30^,^31^,^32^,^33^,^34^. Instead, there is evidence that they can play a role in perceptual categorization^35^. Even in our study, at the population level neurons seem to participate to a categorization process (see Fig. 3c). Note, however, that, at the single neuron level, the majority of them code specifically one stimulus (HS neurons). Concerning non-HS neurons, it is possible that, due to the repetition of the video stimuli sharing similar meanings (e.g. actions performed by a monkey in a first or third person view), they can have undergone to a process of association between these stimuli (see ref. 36). If the task had required a discrimination among the different stimuli or their use to drive specific behaviors, more neurons could have revealed or developed the capacity either to code specific video stimuli or to associate the stimuli in general categories.

### Anatomo-functional relation of VLPF neurons with areas involved in coding biological stimuli

Most of the neurons recorded in the present study prefer biological stimuli, since only very few neurons code object motion. Previous evidence of VLPF single neurons responding to biological stimuli demonstrated responses to the observation of static faces^1^,^2^ or to expressive faces associated to vocalizations^3^,^4^. Imaging studies confirmed these data^37^ and extended the coded biological stimuli to forelimb movements^16^,^38^,^39^. Since in all these studies monkeys were simply required to fixate the stimuli, the responses were not related to the selection/preparation of specific behavioral reactions. Thus, it appears that prefrontal cortex, beyond coding contextual information on object properties, also codes biological stimuli. It is known that the visual information on biological stimuli can be provided to VLPF by the inferotemporal cortex^18^,^40^. A combined fMRI and anatomical study identified anatomically connected inferotemporal and VLPF sectors that are active during action observation^16^. The activated region in VLPF corresponds to its caudalmost part (area 45B). However, the analyses presented in that study were based on regions of interest. A previous fMRI study using the same stimuli identified in VLPF a larger activation, including areas 45A and 46^38^. In line with these data, the action observation neurons recorded in the present study were likely located in areas 45, 46 and 12, that are all strongly connected with the inferotemporal cortex^6^,^7^,^11^,^15^,^41^.

In addition to its connections with inferotemporal cortex, it has recently been demonstrated that specific sectors of area 12 and 46 are directly connected with premotor area F5 and parietal areas PFG and AIP^7^,^15^. A large bulk of studies demonstrated that in these areas there are neurons responding to action observation^22^,^23^,^24^,^25^,^42^. However, in all these studies the visual properties of neurons were tested in a naturalistic setting. Three recent studies investigated neuronal responses to action observation using videos^43^,^44^,^45^, demonstrating that neurons in both ventral premotor area F5 and inferior parietal areas AIP and PFG are activated by videos of monkeys and humans actions, as in the present study. Among these studies, only that by Caggiano *et al*.^43^ compared the neuronal visual responses to the presentation of actions performed by a monkey from different perspectives. Interestingly, both their view-selective premotor neurons and VLPF neurons recorded in the present study showed a preference for the subjective perspective with respect to the third-person view. The different distribution between the two coded views could be related to the fact that the subjective perspective is the most frequently observed during daily life. Indeed since birth the monkey develops a robust experience of the visual image of its own hand in action, thus forming a strong memory of it. It is very likely that this kind of first-person representation is easily recovered from memory when its visual counterpart is presented to the monkey.

### Type of code and role of VLPF neurons responding to videos

The presence of neurons responding to action observation in VLPF raises the question of what their discharge encodes. A first, straightforward hypothesis is that, similarly to inferotemporal cortex, these neurons code a visual representation of the action. A second hypothesis is that they code actions in another type of format, for example in motor terms, as it has already been suggested for parietal and premotor mirror neurons^22^,^24^. A third hypothesis is that they code actions at a more abstract level of representation. The results of the control task, in which different parts of the video were obscured, help to disentangle these hypotheses. Interestingly, only about ten percent of the tested neurons, in agreement with the first, ‘visual’, hypothesis, decreased their response during obscuration. Most tested neurons (2/3) did not change their activity when the action was obscured, suggesting that visual information was not the crucial aspect of their code. A similar behavior was found in ventral premotor area F5^46^, where half of the tested mirror neurons maintained their discharge when the final part of the observed grasping act was hidden. Thus, as suggested in the study of Umiltà *et al*.^46^, the permanence of the response during the obscured phase could be interpreted as the generation of an internal motor representation that includes the action outcome. This interpretation was strongly supported, in the above mentioned study, by the finding that premotor neurons responding to action observation activate also during action execution, thus allowing a direct matching between the visual and the motor description of an act. Interestingly, twelve percent of VLPF neurons responding to action observation and tested for their motor properties are also active during hand action execution, in agreement with this ‘motor’ interpretation. As a further support, the large majority of neurons recorded in VLPF during video presentation respond during the observation of goal-directed actions. A third possible explanation, slightly different from the ‘motor’ one, is that these neurons have the role of maintaining active both the motor and the visual components of action representation in the connected parietal/premotor and inferotemporal areas, respectively. This is compatible with the neurons responses in the control task.

Interestingly, a limited number of neurons tested in the masked condition showed a peculiar behavior, that is their discharge shifted from the first to the second epoch, when the first was obscured. It is possible that these neurons, in order to elicit the activation of the action representation, strongly rely on the actual presence of visual information. Thus, if this information is not available in the first epoch, they use that available in the second one, even if this latter is different in purely visual terms. The fact that the response, depending on the presence or not of visual information, can shift to different phases of the observed action further supports the hypotheses that VLPF neurons can generate action representations.

The type of code of VLPF neurons can be related also to the timing of their discharge. The results of our study show that they can activate in different phases of the observed videos. Considering the timing of the discharge of neurons selective for Epoch 2, this is similar to that usually reported for premotor and parietal mirror neurons: they could code specific aspects of the observed movement (e.g. hand-object interaction). Many VLPF neurons, however, discharge during Epoch 1, also before movement onset. This behavior is particularly interesting, because these neurons could predict the type of action the agent is going to perform and its outcome. In this way, based on the context, the monkey could try to interpret others’ actions even before their beginning and use it for planning its behavior.

Regarding this latter function, in the literature there is general agreement that the responses of prefrontal neurons can play a role in exploiting sensory information for guiding behavioral responses (see refs 47 and 48). In our study, the instruction for the monkey was to simply observe videos, and the type of stimulus presented was not relevant for task accomplishment. Thus, the neuronal responses found in the present study cannot be considered as *directly* related to the selection of a specific behavioral output. It remains, however, to be verified the possible role of these neurons for the monkey behavior. The presence of a large percentage of neurons selective for one stimulus and the fact that the majority of neurons respond to observation of goal-directed actions suggests that different aspects of the observed scene (context, agent, vantage point, etc.), by modulating the neural discharge, could favor the selection of a correlated action and the filtering of not appropriate reactions. For example, the observation of an individual grasping an object can elicit in the observer a competitive or cooperative response depending on the type of object, spatial location and direction of the observed agent’s action, hierarchical state of the agent (dominant, submissive), etc. This interpretation is in line with recent evidence demonstrating that orbitofrontal neurons are sensitive to other monkey’s identity^49^ and lateral prefrontal neurons can represent the prediction of other’s choices^50^. In addition, the fact that most tested neurons do not show a motor-related response, can lead to the hypothesis that their visual discharge, together with the absence of a massive activation of the motor neurons, could signal that the action or the biological movement belongs to another agent. In the motor system, neurons responding during action observation and not during action execution have been described in the ventral premotor^22^, parietal^45^,^51^,^52^ and medial frontal cortex^53^. In particular, Yoshida *et al*.^53^ speculate that neurons with this type of responses (partner-type) ‘represent higher-order, agent related information’. It is possible that part of the VLPF neurons participate to the function of discriminating between self and other.

## Materials and Methods

### Subjects

The experiment was carried out on two female Rhesus monkeys (*Macaca mulatta*, M1, M2) weighing about 4 kg. All methods were performed in accordance with the relevant guidelines and regulations. In particular, the animal handling, as well as surgical and experimental procedures, complied with the European guidelines (2010/63/EU) and Italian laws in force on the care and use of laboratory animals, and were approved by the Veterinarian Animal Care and Use Committee of the University of Parma (Prot. 78/12 17/07/2012) and authorized by the Italian Health Ministry (D.M. 294/2012-C, 11/12/2012). The two animals have been also employed in a previous study on the motor properties of VLPF neurons^27^.

### Training and surgical procedures

The monkeys were first trained to be seated on a primate chair and to familiarize with the experimental setup. At the end of the habituation sessions, a head fixation system (Crist Instruments Co. Inc.) was implanted. Then, they were trained to perform the visual tasks described below. After completion of the training, a recording chamber (32 × 18 mm, Alpha Omega, Nazareth, Israel) was implanted on VLPF, based on MRI scan. All surgeries were carried out under general anesthesia (ketamine hydrocloride, 5 mg/kg, i.m. and medetomidine hydrocloride, 0.1 mg/kg, i.m.), followed by postsurgical pain medication^12^,^27^,^54^.

### Recording techniques and signal acquisition

We employed a multi-electrode recording system (AlphaLab Pro, Alpha Omega Engineering, Nazareth, Israel), that used glass-coated microelectrodes (impedance, 0.5–1 MOhm). The microelectrodes were mounted on an electrode holder (MT, Microdriving Terminal, Alpha Omega) allowing electrodes displacement, controlled by a dedicated software (EPS; Alpha Omega). The MT holder was directly mounted on the recording chamber. Neuronal activity was filtered, amplified and monitored with a multichannel processor and sorted using a multi-spike detector (MCP Plus 8 and ASD, Alpha Omega Engineering). Spike sorting was performed using an Off-line Sorter (Plexon, Inc, Dallas TX, USA).

The experiment was controlled by a homemade Labview software. In particular, digital output signals were used to trigger: the onset and offset of fixation point; the beginning and the end of the visual stimuli presentation; the reward delivery. All these signals were also used as input for the subsequent alignment of the neural activity. Further digital signals consisted in the monkey hand contact with the resting position and the switching on and off of the screen. This latter signal was used to align the neural activity with actual stimulus presentation.

Analog signals provided information about eye position. Eye movements were recorded using an infrared pupil/corneal reflection tracking system (Iscan Inc., Cambridge, MA, USA) positioned above the screen in front of the monkey. Sampling rate was 120 Hz.

### Experimental apparatus

During training and recording sessions the monkeys seated on the monkey chair with the hand contralateral to the hemisphere to be recorded on a resting position. The screen where the visual stimuli were presented was positioned at a distance of 54 cm from monkey’s eyes. The monitor resolution was of 1680 × 1050 pixel and its geometrical center was located at monkey’s eyes level. A laser spot could be projected on the center of the screen as fixation point. A phototransistor was placed on the screen in order to provide the onset and offset of the videoclips presentation.

### Experimental paradigm

In order to evaluate the response of VLPF neurons to observation of visual stimuli, we displayed videos (12° × 12°) showing several biological stimuli and object motion (see below), while the monkey maintained fixation within a 6° × 6° fixation window centered on the video (see also Supplementary information). The sequence of events occurring during each trial is shown in Fig. 1a. Each trial started with the monkey keeping its hand on the resting position, and the fixation point was turned on. The monkey had to fixate it for a randomized time interval (500–900 ms). If fixation was maintained for the requested period, the fixation point was turned off and one of the videoclips was presented (duration 1800 ms). At the end of the video, the fixation point was turned on again for a randomized period (500–900 ms) and the monkey had to keep fixation on it. The trial was accepted as correct, and the monkey was rewarded if it kept its eyes within the fixation window for the duration of each phase of the task, and it did not release the hand from the resting position. Discarded trials were repeated at the end of the sequence in order to collect at least 10 presentations for each stimulus. The order of stimuli presentation was randomized.

A subset of neurons was further tested with control tasks. The described procedures were the same for both the basic and the control tasks.

### Stimuli

In the basic task, the monkey was required to passively observe 6 different videos, in which the stimulus was presented in the central part of the screen. The construction of the set of video stimuli was devised to present to the monkey goal-related or non-goal-related actions, different agents and presence or absence of an object. Specifically, the 6 stimuli were the following (see Fig. 1b).

#### Monkey grasping in first person perspective (MGI)

A monkey right forelimb enters into the scene, reaches and grasps a piece of food located at the center of the video, lift it and bring it toward herself (only the initial phase of this latter movement is visible). The observed forelimb is presented as if the observing monkey was looking at its own forelimb during grasping.

#### Monkey grasping in third person perspective (MGIII)

A monkey, located in front of the observer, with its left forelimb reaches and grasps a piece of food located at the center of the video, and brings it toward herself.

#### Human grasping (HG)

A human actor, located on the right of the video reaches, grasps and lifts an object, located at the center of the video, with his right forelimb.

#### Human mimicking (HM)

A human actor, located on the right of the video, pantomimes the same action shown in HG, without the object.

#### Biological movement (BM)

A human actor located on the right of the video extends his right forelimb with the hand open, to reach the central part of the screen. No object is present.

#### Object motion (OM)

An object is presented in the center of the screen and moves following the same trajectory as in HG. This stimulus was obtained by removing the agent from HG, in order to have same kinematics of the object as in HG.

In the videos the agents’ faces were not shown in order to avoid the possible influence of neural responses due to face presentation. Note that the agent’s effector enters from the side contralateral to the monkey recorded hemisphere.

In the control tasks, the monkey was required to passively observe 12 stimuli, representing either human or monkey actions. Four of them were the same as in the basic task (MGI, MGIII, HG and HM), while the other 8 stimuli consisted in their modified versions (Fig. 1c). In these versions, we obscured the first or the second phase, using a black shading. The shading of the first part of the video prevents the observation of the target/context and the beginning of forelimb movement, while the shading of the second part obscures actual grasping (or its mimicking). This control task was aimed at assessing which visual features were crucial to elicit neurons responses.

### Data analysis

In the basic task, the neural activity was recorded for at least 60 successful trials. For the statistical analysis three epochs were defined:

(1) Baseline: 500 ms before the beginning of the videos, during which the monkey was looking at the fixation point; (2) Video Epoch 1: the first 700 ms of the videos (except for MGI, where, because of the fastest arm movement, the epoch lasted 500 ms); (3) Video Epoch 2: the subsequent 700 ms. Note that Epoch 1 includes the context of the scene and the beginning of the forelimb movement, while Epoch 2 includes the hand-object contact and the end of the action/pantomime (in all videos except OM) and the actual object motion (in OM).

Single-neuron responses were statistically evaluated by means of a 3 × 6 ANOVA for repeated measures (Factors: Epochs, Stimuli, p < 0.01) followed by Newman–Keuls post hoc tests. Since trials were randomized, changes of the baseline activity across trials were not expected. For this reason, 3 neurons showing significant differences between the baselines of the stimuli were excluded from the database.

A neuron was included in the dataset when the 3 × 6 ANOVA revealed: 1) a significant Main Effect Epoch and, in the Post-hoc test, a significant difference between at least one of the two video epochs and the baseline and/or 2) a significant Interaction effect, in which the Post-hoc test showed a significant difference between at least one of the video epochs of one stimulus and its baseline.

Out of the neurons tested with the control tasks, those that responded to HG and/or HM in the basic task were analyzed also for their responses during observation of the masked stimuli depicting the human agent, while those responsive to MGI or/and MGIII were analyzed also for their responses to the masked stimuli depicting the monkey agent. The activity recorded during the observation of the stimuli presented in the control task was analyzed with a 3 × 2 ANOVA for repeated measures (Factors, Stimuli: Original, Shaded first part and Shaded second part; Epochs: 1 and 2, p < 0.01).

### Computation of depth of tuning, selectivity and category indexes

To quantify the degree and depth of selectivity of the neurons for the video stimuli, we calculated two indexes: the depth of tuning index (d_i_), measuring the difference between the maximal and the minimal response normalized to the cell’s maximal response, and the selectivity index (s_i_) quantifying the extent to which activity in all non-preferred stimuli deviates from the maximal activity for the preferred stimulus. Each index was computed for each neuron across the six video stimuli employed in the basic task. These indexes, previously employed for assessing the neuronal directional tuning^26^, are defined as follow:


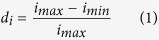



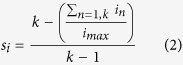


where *k* is the number of video stimuli; and *i_min_* and *i_max_* are, respectively, the minimum and maximum responses of the *i^th^* neuron across the six different video stimuli. Note that both indexes range from 0 to 1. Thus, a d_i_ of 1 means that a neuron does not fire at all above baseline in the worst condition, a s_i_ of 1 means that a neuron responds only to one video stimulus. A d_i_ or an s_i_ of 0 indicates that the firing rate is the same for all videos. This latter situation, however, is absent in our dataset, since we included only neurons having some type of selectivity. Thus both indexes are always higher than 0.

In order to evaluate whether the neurons selective for at least one video stimulus could be classified in the general categories described above, for each excitatory neuron (n = 99) we calculated three category indexes indicating to which extent a neuron is tuned to goal-related vs. non-goal-related, monkey-related vs. human-related and object-related vs. non-object-related stimuli, respectively. First, we calculated the normalized mean activity^27^ for each neuron in its preferred epoch. Then, we summed the mean activity recorded for each video stimulus belonging to the same category, multiplied the result by the number of video stimuli included in that category, and divided by the total number of video stimuli, thus obtaining the mean activity for each category (e.g. goal-related mean activity = (MGI + MGIII + HG)*3/6). This procedure allowed us to compare categories represented by a different number of video stimuli. Finally, we subtracted, from the mean activity of one category, that of the opposite one: goal minus non-goal; monkey minus human; object minus non-object. The result of this operation is an index varying from 1 (absolute preference for one category) to −1 (absolute preference for the other). The value of 0 indicates that a neuron does not show preference for one of the two considered opponent categories.

These category indexes have been used to plot the coordinates of each neuron activity in a 3D space representing its relatedness to the three pairs of the above mentioned categories. The standard K-means clustering algorithm (Matlab) was applied in this 3D space for various numbers of clusters (ranging from 2 to 6). The ‘silhouette’ function in Matlab provided a measure of the clusters separation. The silhouette values range from +1 to −1; +1 denote clear cluster assignment and −1 marks points with questionable cluster assignment. A successful clustering has mean silhouette value greater than 0.6. The metric used in both K-means and silhouette was squared Euclidean distance.

## Additional Information

**How to cite this article:** Simone, L. *et al*. Action observation activates neurons of the monkey ventrolateral prefrontal cortex. *Sci. Rep.* **7**, 44378; doi: 10.1038/srep44378 (2017).

**Publisher's note:** Springer Nature remains neutral with regard to jurisdictional claims in published maps and institutional affiliations.

## Supplementary Material

Supplementary Information

## Figures and Tables

**Table 1:** Number of neurons showing stimulus preference (HS) and relative percentage.

Stimulus	n. neurons	% neurons
MGI	31	40%
MGIII	15	19%
HG	9	12%
HM	11	14%
BM	9	12%
OM	2	3%
Total	77	100%

Neurons are classified on the basis of the stimulus producing the statistically highest response (3 × 6 ANOVA, followed by Newman-Keuls post hoc test, p < 0.01).
