# Supplementary material for: Action observation activates neurons of the monkey ventrolateral prefrontal cortex
Source: Sci Rep. 2017 Mar 14;7:44378. doi: 10.1038/srep44378 (PMC5349536; doi:10.1038/srep44378)

Action observation activates neurons of the monkey ventrolateral prefrontal cortex.

Luciano Simone[1,2], Marco Bimbi[1], Francesca Rodà[1], Leonardo Fogassi[1] and Stefano Rozzi[1*]

[1]Department of Neuroscience, University of Parma, via Volturno 39, 43125 Parma, Italy.

[2]Dipartimento di Scienze biomediche e chirurgico specialistiche, Università di Ferrara, via Fossato di Mortara 64/A 44121, Ferrara, Italy

***Corresponding author:**

Stefano Rozzi

Dipartimento di Neuroscienze, Università degli Studi di Parma

Via Volturno 39, 43125 – Parma – Italy

e-mail: stefano.rozzi@unipr.it

FAX: ++39-0521-903900

Phone: ++39-0521-903879

Supplementary material

Analysis of the oculomotor behavior

In order to evaluate the monkeys oculomotor behavior during the behavioral tasks within the imposed 6°x6° fixation window, we analyzed the horizontal and vertical components of the eye position acquired during recordings. Eye horizontal and vertical coordinates are expressed in terms of eccentricity (in degrees) from the fixation point, located at the center of the videos frames. In particular, for each monkey, we analyzed the eye traces recorded during correct trials in the sites in which neurons included in the database were recorded. Trials in which blinking occurred were excluded from the analysis. For each monkey and video, we calculated the mean and standard deviation of horizontal and vertical eye coordinates during a time period encompassing both Video epoch 1 and 2.

Behavioral results

We analyzed 876 trials from M1 and 911 from M2. The results indicate that, although the task required a 6°x6° fixation window, both monkeys, during the presentation of each video fixated within a much narrower spatial window, usually smaller than 2°x2°. More in details, M1 kept her eyes in the following positions (calculated in degrees from the center of the video). Horizontal component. HG: -0.056 ± 0.59; HM: -0.48 ± 0.78; MGI: -0.46 ± 0.52; MGIII: -0.21 ± 0.5; BM: -0.05 ± 1.2; OM: -0.5 ± 0.52. Vertical component. HG: -0.95 ± 0.79; HM: -0.58 ± 0.99; MGI: -0.93 ± 0.90; MGIII: -0.62 ± 0.91; BM: 0.24 ± 0.87; OM: -0.51 ± 0.85. M2 kept her eye in the following positions. Horizontal component. HG: -0.072 ± 0.5; HM: -0.65 ± 0.64; MGI: -0.73 ± 0.53; MGIII: -0.33 ± 0.38; BM: -0.28 ± 0.66; OM: -0.65 ± 0.45. Vertical component. HG: -0.12 ± 0.60; HM: -0.13 ± 0.79; MGI: -0.05 ± 0.82; MGIII: 0.16 ± 0.73; BM: 0.62 ± 1.03; OM: -0.08 ± 0.71.

Supplementary Figure 1. Eye position during the observation of the basic task stimuli.

A. The black squares represent, for each monkey, the 12x12° space sector in which videos are presented, centered on the location of the fixation point (0,0). The colored circles and rectangles represent the mean and the standard deviation, respectively, of the position of the monkeys' eyes during the presentation of the six videos employed in the basic task (Video Epoch 1 and 2). Although the task imposed a 6°x6° fixation window, both monkeys fixated each video within a much narrower spatial window, generally smaller than 2°x2°.

B. The required 6°x6° fixation windows (yellow square) and the actual mean ± standard deviation of the eye position (red dots and rectangles, respectively) of M1 are plotted on representative frames (12x12°) of the first and second Video Epochs.

Supplementary figure. Eyes position during the observation of the basic task stimuli

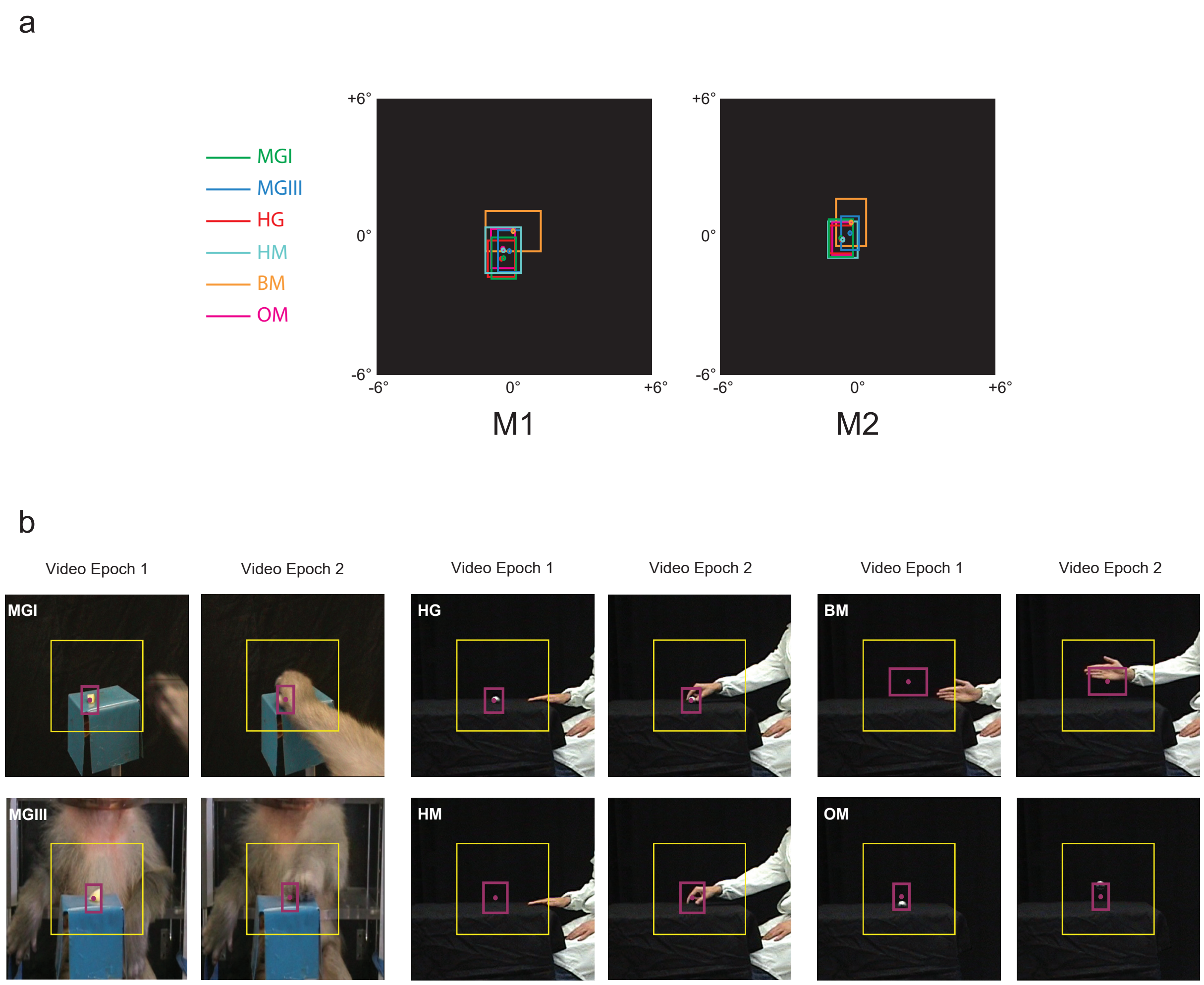

Supplement: Supplementary Information [file srep44378-s1.pdf]
